# Is dengue and malaria co-infection more severe than single infections? A retrospective matched-pair study in French Guiana

**DOI:** 10.1186/1475-2875-11-142

**Published:** 2012-05-01

**Authors:** Loïc Epelboin, Matthieu Hanf, Philippe Dussart, Sihem Ouar-Epelboin, Félix Djossou, Mathieu Nacher, Bernard Carme

**Affiliations:** 1 CIC-EC Antilles Guyane CIE 802 Inserm, Centre Hospitalier Andrée Rosemon, Cayenne, French Guiana; 2 Research team EPaT EA 3593, University of French West Indies and French Guiana, Cayenne, French Guiana; 3 Service de Maladies Infectieuses et Tropicales, Centre Hospitalier Pitié-Salpêtrière, 47–83 bd de l’Hôpital, 75013, Paris, France; 4 Centre National de Référence des Arboviroses, Institut Pasteur de Guyane, Cayenne, French Guiana; 5 Infectious and Tropical Diseases Department, Centre Hospitalier Andrée Rosemon, Cayenne, French Guiana

**Keywords:** Dengue, Malaria, French Guiana, Thrombocytopaenia, Case–control studies

## Abstract

**Background:**

Dengue and malaria are two major arthropod-borne infections in tropical areas, but dual infections were only described for the first time in 2005. Reports of these concomitant infections are scarce and there is no evidence of more severe clinical and biological pictures than single infections.

**Methods:**

To compare co-infections to dengue alone and malaria alone, a retrospective matched-pair study was conducted between 2004 and 2010 among patients admitted in the emergency department of Cayenne hospital, French Guiana.

**Results:**

104 dengue and malaria co-infection cases were identified during the study period and 208 individuals were matched in two comparison groups: dengue alone and malaria alone. In bivariate analysis, co-infection clinical picture was more severe than separated infections, in particular using the severe malaria WHO criteria. In multivariate analysis, independent factors associated with co-infection versus dengue were: masculine gender, CRP level > 50 mg/L, thrombocytopaenia < 50 10^9^/L, and low haematocrit <36% and independent factors significantly associated with co-infections versus malaria were red cells transfusion, low haematocrit < 36%, thrombocytopaenia < 50 10^9^/L and low *Plasmodium* parasitic load < 0.001%.

**Conclusions:**

In the present study, dengue and malaria co-infection clinical picture seems to be more severe than single infections in French Guiana, with a greater risk of deep thrombocytopaenia and anaemia.

## Background

Dengue fever and malaria are the most common arthropod-borne diseases in humans and represent major public health problems. Dengue virus (family *Flaviridae*, genus *Flavivirus*) and *Plasmodium* parasites are widespread in American and Asian tropical regions and their endemic areas overlap extensively. Nevertheless, reports of malaria and dengue dual infection are scarce. Since the first case reported in 2005 [1], only case-reports and two descriptive studies have been published. They have been reported with *Plasmodium falciparum* and/or *Plasmodium vivax* in India and Pakistan [2-5], Southeast Asia [6,7], French Guiana [8] and Brazil [9]. This phenomenon seems to be uncommon. In a study performed in Thailand among 194 patients with dengue, no co-infection with malaria was found [10], but in French Guiana, a retrospective study performed in 2004–2005 on 1,723 consecutive febrile emergency patients found 17 co-infections, including six acute concurrent infections (e.g. 1% of dengue and 4% of malaria cases) [8]. The influence of co-infections on severity is not straightforward, therefore, the aim of this study was to differentiate clinical and biological picture of co-infections from infections alone and determine whether patients infected by both malaria and dengue (MD) were more severe than either infection alone (respectively M and D).

## Methods

### Study location

French Guiana is a French Overseas territory located on the north-eastern coast of South America. About 90% of its surface of 84,000 km^2^ is Amazonian rain forest; the remaining 10% in the north is a coastal plain where 90% of the 215,000 inhabitants live and Cayenne and surroundings contain almost 50% of the population in 2009 [11]. Malaria and dengue fever (DF) represent two major public health concerns in French Guiana. Malaria is endemic and the annual number of cases ranges from 3,200 to 4,700 [12]. Until 2006, *P. vivax* represented 50% of annual cases. The current proportion of *P. vivax* malaria is 75%, as in the rest of the Americas [12-14]. Since the first cases of DF were reported in French Guiana in 1943, an increase in the number of DF cases and DF outbreaks and the emergence of dengue hemorrhagic fever (DHF) have been observed [15]. All four dengue virus serotypes circulate in French Guiana. The last two mains epidemics occurred in 2006 and 2009, and dengue is currently endemic. Until 2005, dengue outbreaks were exclusively described on the coast. Since 2006, outbreaks of DF have been reported in interior villages where malaria is endemic [16].

### Study population

A matched retrospective study was conducted comparing patients infected with concurrent malaria and dengue to patients with either infection alone. The study population included all patients admitted in the emergency department of Cayenne hospital, between June 2004 and February 2010. The diagnosis of dengue and malaria co-infection was made on the basis of concomitant biological diagnosis of dengue and malaria within seven days in patients with a compatible clinical picture. Two control groups were constituted: the group M with positive biological diagnosis for malaria and negative for dengue, according to the criteria defined in the next paragraph, and the contrary for the group D. Control cases were matched on the date of biological diagnosis of infection.

Case definitions were based on compatible clinical history and biological diagnosis. Malaria diagnosis relied on the identification of haematozoa on a thin blood film and/or on a thick blood film stained with Giemsa (group MD and M). The screening sensitivity was ≈ 6 plasmodia/μL (1/1,000 leukocytes). The asexual parasite load (PL) was classified in five classes: class 5: >1.25%; class 4: 0.125 to 1.25%; class 3 : 0.0125% to 0.125%; class 2: 0.00125 to 0.0125%; and class 1: ≤0.00125. Malaria rapid diagnosis tests were not systematically performed on the study period. Due to the evolution of the techniques between 2004 and 2010, the laboratory diagnosis of dengue relied on different methods. Direct diagnosis was based on virus isolation, genome detection by Reverse Transcriptase-Polymerase Chain Reaction (RT-PCR) or NS1 antigen detection introduced in 2006 in French Guiana. Indirect diagnosis was based on detection of specific anti-dengue IgM and/or IgA antibodies in patients’ sera [17]. When NS1 antigen detection was available, RT-PCR, which allows serotype identification, was not systematically performed.

Concerning dengue definition (groups MD and D), cases were separated in two groups: “confirmed acute dengue cases” (CADC) were defined by direct biological diagnosis (NS1 antigen and/or RT-PCR and/or virus isolation), IgM seroconversion (early serum sample negative for IgM but convalescent sample positive) or IgA antibodies detection. “Likely dengue cases” (LDC) were defined by IgM antibodies detection. Indeed, IgM appear between the 3^rd^ and 5^th^ day of fever but can persist for over three months and IgA appear concomitantly with IgM but does not persist longer than five to six weeks [18,19]. There was no discrimination between dengue primary infection and secondary infection, e.g., further infection(s) by dengue of a different serotype.

### Covariates included, data collection and statistical analysis

Patients’ data, including socio-epidemiologic data, previous medical history, clinical symptoms, and biological results, were obtained from the computerized medical charts. Data were analysed using R version 2.10.0® and the Epicalc® package.

The continuous variables of interest were categorized following the laboratory cut-off values, or published values. They generally were dichotomized because of the small sample size.

Two analyses were performed comparing separately MD to D and MD to M. For categorical variables, a matched bivariate analysis using the Wald test was performed to identify factors associated with co-infections. Statistical significance was set at p <0.05. Variables with a p-value < 0.2 in bivariate analyses were entered into multivariate model to identify the factors independently associated with dengue-malaria co-infections. As bivariate analysis were made in an exploratory way and used as a selection criterion for inclusion in the final multivariate model (p < 0.20), we did not judge as a necessity to adjust bivariate p-values for multiple comparisons. Thus, bivariate p-values near to 0.05 must be relativized. To obtain more powerful models, and because of the missing data inherent to retrospective studies, variables obtained from anamnesis and clinical examination and variables with more than 5% of missing data were excluded from the model. Thus, conditional multivariate backward stepwise logistic regression estimated the adjusted *odds ratio* (OR) and the confidence intervals linked to co-infections.

### Ethical considerations

The retrospective use of anonymous patient files on the site of patient care is authorized by the French National Commission on Informatics and Liberties. All the data collected retrospectively were anonymized in a standardized case report form and in the database.

## Results

### Cases description

**Figure 1:**
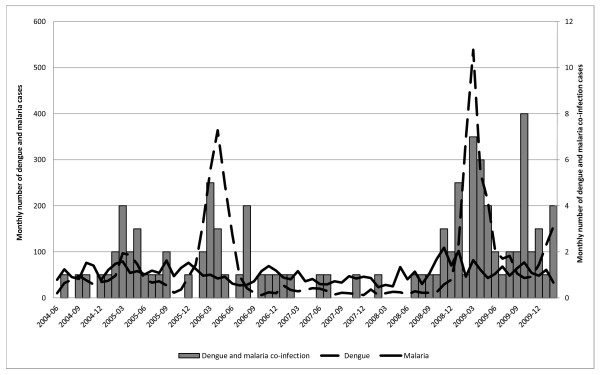
**Monthly cases of malaria, dengue fever (curves) and dengue and malaria co-infection (columns) in the Emergency department of Cayenne Hospital, French Guiana, between June 2004 and February 2010.** Source : Institut de Veille Sanitaire - CIRE Antilles-Guyane, Reference National Laboratory of Arboviruses and Parasitology–Mycology Unit, Cayenne Hospital, French Guiana.

Between June 2004 and February 2010, 104 patients satisfied the criteria for MD (Figure 1). Consequently, 208 patients were matched in each comparison group. Among the 104 MD patients, 75 (72.1%) were men and 11 (10.6%) were 15 years-old or under. The mean age was 33.8 years (range: 6 months to 83 years). Forty-one (39.4%) were considered as CADC and 63 (60.6%) as LDC versus 150 (72.1%) and 58 (27.9%; *p* <0.001) in the group D, respectively (Table 1). The dengue virus serotype could be identified for only 10 (9.6%) of co-infected patients (DENV-1: 3 (30%); DENV-2: 2 (20%) and DENV-3: 5 (50%)) and 91 in the control group (DENV-1: 25 (27.5%); DENV-2: 25 (27.5%); DENV-3: 28 (30.8%) and DENV-4: 13 (14.2%)). No significant difference was found in the proportion of *P. vivax* between the MD group and M group: *P. vivax* (76.7% vs. 68.1% respectively), *P. falciparum* (20.4% vs. 28%) and association of *P. vivax* and *P. falciparum* (2.9% vs. 3.9%). Species identification was not possible in two patients because they had received anti-malarial treatment after positive rapid diagnostic testing in a health centre. A low PL (class 1 and 2) tended to be more frequent in the MD group than M group (p 0.08) (Table 2).

**Table 1:** Laboratory diagnosis of dengue infection in the co-infected group and in the single dengue infection group

**Dengue laboratory diagnosis**	**Co-infection** **n (%)**	**Dengue** **n (%)**	**Cases**
Cell culture virus isolation and/or RT-PCR*	9 (8.7)	56 (26.9)	CADC^§^
NS1 antigen +/− IgM*	11 (10.6)	84 (40.4)	
IgM + IgA*	14 (13.5)	9 (4.3)	
IgM seroconversion	7 (6.7)	1 (0.5)	
IgM	63 (60.6)	58 (27.9)	LDC^§^
Total	**104**	**208**	

*RT-PCR Reverse transcriptase polymerase chain reaction, *IgM* Anti dengue immunoglobulin M, *IgA* Anti dengue immunoglobulin A.

^§^*CADC* Confirmed acute dengue cases, *LDC* Likely dengue cases.

**Table 2:** Matched bivariate analysis between co-infected patients and pure malaria infected patients

Results n (%)	**Co-infection** **(n = 104)**	**Malaria** **(n = 208)**	p**
**Males**	75 (72.1)	162 (77.9)	0.26
**Children (<15 years-old)**	11 (10.6)	18 (8.7)	0.58
**Journey in forest**	26 (25)	51 (24.6)	0.96
**Inhabitant of the coast**^**¥**^	86 (83.5)	151 (74.4)	0.11
**Long admission (≥2 days)**	43 (41.3)	72 (34.6)	0.25
**Admission**	37 (35.6)	69 (33.2)	0.67
**Medical history of malaria**	52 (50)	115 (62.8)	0.02^§^
**Time since last malaria attack < 90 days**	19 (55.9)	49 (51.6)	0.91
**Medical history of dengue**	3 (3.4)	16 (9)	0.08^§§^
**Duration of fever > 5 days**	48 (48.5)	61 (29.9)	0.001^§^
**Red cells transfusion**	8 (7.7)	3 (1.4)	0.01^§^
**Tachycardia (>90 bpm)**	79 (79)	165 (82.1)	0.64
**Hypotension (<90 mmHg)**	10 (10.4)	7 (3.6)	0.01^§^
**Fever ≥ 40°C**	15 (14.7)	24 (11.9)	0.47
**Retro-orbital pain**	4 (3.9)	10 (5)	0.61
**Chills**	36 (35.6)	64 (32.2)	0.7
**Hemorrhagic signs**	12 (11.5)	25 (12.4)	0.81
**Shock signs**	7 (6.8)	8 (4)	0.16^§§^
**Rash**	1 (1)	5 (2.5)	0.4
**Nausea and / or vomiting**	58 (56.9)	93 (45.8)	0.06^§§^
**Neurological disorders**	7 (6.8)	5 (2.5)	0.08^§§^
**Dehydration**	7 (7.4)	10 (5)	0.37
**Mucocutaneous pallor**	11 (11.6)	7 (3.5)	0.02^§^
**Splenomegaly**	9 (12.3)	23 (18.3)	0.08^§^
**Mucocutaneous jaundice**	9 (8.7)	9 (4.3)	0.14^§§^
**ENT symptoms**	6 (5.8)	10 (4.8)	0.72
**Severe “malaria” cases††**	34 (32,7)	39 (18,7)	0,007
**Low parasitaemia (class < 3)**	20 (19.2)	24 (11.5)	0.08^§§^
**Mildly low hemoglobin (<12 g/dl)**	37 (35.6)	43 (20.7)	0.05^§§^
**Low hematocrit (<36%)**	43 (41.3)	49 (23.6)	0.002^§^
**Deep thrombocytopenia (<50 G/L)**	23 (22.1)	25 (12)	0.02^§^
**Low prothrombin (<70%)**	14 (21.2)	22 (15.4)	0.33
**Hyponatremia (<130)**	7 (7.1)	15 (7.4)	0.87
**Hypokalemia (<3)**	6 (6.1)	15 (7.4)	0.67
**Elevated creatinin (>111 μmol/L)**	4 (4)	14 (7)	0.29
**Elevated bilirubin (>50 μmol/L)**	16 (16.8)	20 (10.5)	0.06
**SGPT (>2 N)**	10 (10.2)	33 (16.7)	0.16^§^
**SGOT >2 N**	13 (13.3)	23 (11.6)	0.66
**CRP <5**	2 (2)	1 (0.5)	0.22
** CRP 5 à 50**	29 (29)	78 (38)	-
** CRP > 50**	69 (69)	126 (61.5)	-

* *p* calculated with Wald test in matched bivariate analysis.

^**§**^ Significant *p*-values < 0.05.

^**§§**^*p*-values < 0.2 included in the matched multivariate model.

*bpm* Beats per minute.

^**¥**^ Inhabitants of the coast are defined as people living in Cayenne, Rémire-Montjoly, Matoury Macouria, Kourou, Irakoubo or Mana.; Other people declared to live in Cacao, Roura, Montsinnéry-Tonnégrande, Régina, Saül, St-Elie, Saint Laurent du Maroni, Apatou, Maripasoula, Papaïchton, St George de l’Oyapock, Camopi or Trois-Sauts. 4 people came from France, 1 from the French Caribbean and 1 from French Polynesia.

^††^ WHO 2000 criteria for severe *P. falciparum* malaria

*ENT* Ear, Nose and Throat, *SGPT* Serum Glutamopyruvate Transferase, *SGOT* Serum Glutamooxaloacetate Transférase, *CRP* C-reactive protein.

### Comparison of co-infection with dengue

Clinical and biological pictures of co-infection cases were different from single infections and bivariate comparisons showed more differences between MD and D than between MD and M (Table 3). MD patients were more often adult men. A quarter of them reported having recently visited the forest (military, forest workers or gold miners) versus 3% in the D group. Patients from MD group resided more frequently far from the coast than D patients and had a history of malaria and recent malaria attacks (<3 months). The duration of fever was longer in MD patients and they were hospitalized more frequently than D patients, but hospitalization was not longer. More patients required a transfusion in the MD group. The clinical presentation in MD patients was generally more severe, with more fever above 40°C, tachycardia, initial hypotension, nausea, vomiting and dehydration than D patients. Furthermore, cases which fulfilled one or more of the WHO clinical and/or biological criteria for severe falciparum malaria [20] were more frequent in the MD group than in the D group (p < 0.001). Increased C - reactive protein (CRP), especially > 50 mg/L, was significantly associated with MD co-infections (*p* <0.001) relative to dengue alone. Retro-orbital pain, skin rash and ENT symptoms were significantly associated with D. Anaemia (p 0.02), severe thrombocytopaenia (p <0.001), and elevated bilirubin (p <0.001) were more frequent in MD patients. CADC diagnoses were significantly more frequent in the D group than in the MD group. Anaemia, severe thrombocytopaenia, male gender, high CRP level and LDC diagnosis were significantly associated with co-infection in multivariate analysis (Table 4).

**Table 3:** Matched bivariate analysis between co-infected patients and pure dengue infected patients

Results n (%)	**Co-infection (n = 104)** **N (%)**	**Dengue (n = 208)** **N (%)**	p*
**Males**	75 (72.1)	119 (57.2)	0.01^§^
**Children (<15 years-old)**	11 (10.6)	49 (23.6)	0.008^§^
**Journey in forest**	26 (25)	7 (3.4)	<0.001^§^
**Living on the coast**^**¥**^	86 (83.5)	183 (92.9)	0.025^§^
**Long admission (≥2 days)**	43 (41.3)	71 (34.1)	0.23
**Admission**	37 (35.6)	45 (21.6)	0.01^§^
**Medical history of malaria**	52 (50)	21 (10.2)	<0.001^§^
**Time since last malaria attack < 90 days**	15 (44.1)	0 (0)	-
**Medical history of dengue**	3 (3.4)	5 (2.7)	0.64
**Duration of fever > 5 days**	48 (48.5)	33 (16.3)	<0.001^§^
**Red cells transfusion**	8 (7.7)	2 (1)	0.03^§^
**Tachycardia (>90 bpm)**	79 (79)	128 (64.6)	0.03^§^
**Hypotension (<90 mmHg)**	10 (10.4)	3 (1.8)	0.02^§^
**Fever ≥ 40°C**	15 (14.7)	10 (4.9)	0.006^§^
**Retro-orbital pain**	4 (3.9)	26 (12.6)	0.01^§^
**Chills**	36 (35.6)	37 (18)	0.002^§^
**Hemorrhagic signs**^†^	12 (11.5)	32 (15.5)	0.31
**Shock signs**^‡^	7 (6.8)	5 (2.4)	0.06^§§^
**Rash**	1 (1)	30 (14.6)	0.007^§^
**Neurological disorders**	7 (6.8)	8 (3.9)	0.28
**Nausea and / or vomiting**	58 (56.9)	94 (45.6)	0.07^§§^
**Dehydration**	7 (7.4)	4 (1.9)	0.04^§^
**Mucocutaneous pallor**	11 (11.6)	3 (1.5)	0.003^§^
**Splenomegaly**	9 (12.3)	3 (3.1)	0.04^§^
**Jaundice**	9 (8.7)	6 (2.9)	0.03^§^
**ENT symptoms**	6 (5.8)	42 (20.2)	0.002^§^
**Severe “malaria” cases**^††^	34 (32,7)	33 (15,9)	<0,001
**Confirmed acute dengue cases**	41 (39.4)	150 (72.1)	<0.001^§^
**Mildly low hemoglobin (<12 g/dl)**	37 (35.6)	40 (19.2)	0.002^§^
**Low hematocrit (<36%)**	43 (41.3)	34 (16.4)	<0.001^§^
**Deep thrombocytopenia (<50 G/L)**	23 (22.1)	6 (2.9)	<0.001^§^
**Low prothrombin (<70%)**	14 (21.2)	19 (13.9)	0.18^§§^
**Hyponatremia (<130)**	7 (7.1)	5 (2.5)	0.08^§§^
**Hypokalemia (<3)**	6 (6.1)	3 (1.5)	0.04^§^
**Elevated creatinin (>111 μmol/L)**	4 (4)	4 (2)	0.3
**Elevated bilirubin (>50 μmol/L)**	16 (16.8)	3 (1.6)	<0.001^§^
**SGPT (>2 N)**	10 (10.2)	32 (16)	0.2
**SGOT >2 N**	13 (13.3)	41 (20.5)	0.16^§§^
**CRP <5**	2 (2)	73 (35.3)	<0.001^§^
**CRP 5 à 50**	29 (29)	115 (55.6)	-
**CRP > 50**	69 (69)	19 (9.2)	-

* *p* calculated with Wald test in matched bivariate analysis.

^**§**^ Significant *p*-values < 0.05.

^**§§**^*p*-values < 0.2 included in the matched multivariate model

*bpm* Beats per minute..

^**¥**^ People living on the coast are defined as people living in Cayenne, Rémire-Montjoly, Matoury Macouria, Kourou, Irakoubo or Mana. ; Other people declared to live in Cacao, Roura, Montsinnéry-Tonnégrande, Régina, Saül, St-Elie, Saint Laurent du Maroni, Apatou, Maripasoula, Papaïchton, St George de l’Oyapock, Camopi or Trois-Sauts. 4 people came from France, 1 from the French Caribbean and 1 from French Polynesia.

^**†**^Hemorrhagic signs included purpura, gingival bleeding, epistaxis, macroscopic hematuria, hematemesis, melena, rectal bleeding and hemoptysis.

^**‡**^Signs of shock included systolic blood pressure <90 mmHg or not responding to IV fluids and with or without oliguria. ENT symptoms included diagnoses of rhinitis, nasopharyngitis, pharyngitis, sinusitis and otitis.

^††^ WHO 2000 criteria for severe *P. falciparum* malaria [20].

*ENT* Ear, Nose and Throat, *SGPT* Serum Glutamopyruvate Transferase, *SGOT* Serum Glutamooxaloacetate Transférase, *CRP* C-reactive protein.

**Table 4:** Results of bivariate and multivariate analysis of co-infected group and single dengue infection and single malaria infection respectively

	**Outcome**	**Bivariate analysis**			**Multivariate analysis**			**Sensitivity (%)**	**Specificity** **(%)**
		OR*	95% CI*	p*	OR**	95% CI**	p**		
Co-infection versus dengue	**Confirmed acute dengue cases**	5.3	2.9-9.7	**<0.001**	**4.5**	**1.7-11.9**	**0.003**	79.1	52.1
	**Males**	1.9	1.2-3.2	0.01	3.4	1.1-10.3	**0.03**	38.7	75.4
	**Low hematocrit (<36%)**	4.3	2.3-8.2	<0.001	8.4	2.2-32-5	**0.002**	53.5	74.3
	**Deep thrombocytopenia (<50. 10**^**9**^**/L)**	13.8	4.1-46	<0.001	11.7	1.7-79.2	**0.01**	79.3	71.4
	** CRP 5 to 50 mg/L**	5.0	1.1-21.7	<0.001	5.3	0.9-29.3	0.06	-	-
	** CRP >50 mg/L**	75.4	16-356.7	<0.001	74.4	12.2-453.3	<**0.001**	78.4	85.8
Co-infection versus malaria	**Red cells transfusion**	7.3	1.5-34.7	0.01	5.3	1.04-26.7	**0.04**	72.7	68.1
	**Low hematocrit (<36%)**	2.2	1.3-3.6	0.002	2	1.2-3.5	**0.009**	46.0	72.6
	**Deep thrombocytopenia (<50. 10**^**9**^**/L)**	2.2	1.1-4.1	0.02	2.1	1.02-4.1	**0.04**	47.9	69.3
	**Low parasitic load (class < 3)**	1.8	0.9-3.3	0.08	2.2	1.08-4.3	**0.03**	45.4	68.6

* *Odds ratio* (OR) and 95% confidence interval (95% CI) and p calculated by matched bivariate analysis.

** *Odds ratio* and 95% confidence interval and p calculated by matched multivariate analysis.

*CRP* C-reactive protein.

### Comparison of co-infection with malaria

No significant difference between MD and M was found in terms of gender, age, place of residence and forest-related activities (Table 2). A history of malaria was more frequent in the M group. The fever duration was longer in MD patients but not hospitalization. They received significantly more transfusions (p 0.02). Low blood pressure, signs of shock, pallor were significantly associated with the MD group. Anaemia and severe thrombocytopaenia were also significantly more frequent in the MD group. Cases which fulfilled one or more of the WHO criteria for severe falciparum malaria [20] were more frequent in the MD group than in the M group (p 0.007). Anaemia, severe thrombocytopaenia, low parasitaemia, and a high number of blood transfusions were independently associated with co-infections in multivariate analysis (Table 4).

## Discussion

The unexceptional nature of the association of dengue and malaria is confirmed in French Guiana. In regions where these infections are transmitted in close proximity, the classical concept that malaria occurs in rural areas and dengue in urban areas may thus also be contradicted by facts in many countries and simultaneous infections may result from the overlap of the mosquito biotopes [16].

This study presented some minor biases. There were a higher number of cases based on the IgM detection in the MD group than in the D group. Co-infected patients with LDC were tested separately, so the association between MD and thrombocytopaenia, anaemia and frequent transfusions persisted in bivariate analysis, but not in multivariate analysis. However, when testing separately all patients with CADC diagnosis, no significant difference was found in bivariate and multivariate analysis which is probably due to the loss of power. The separation in two groups, LDC and CADC is arbitrary since the decision to perform the direct diagnosis relied on non verifiable information provided by patients on fever duration, a relatively unreliable answer given the frequent linguistic difficulties in FG. Studying confirmed acute cases alone was questionable, and the authors decided to study together likely and confirmed acute cases because it allowed a larger sample. Furthermore, associating likely cases would have minimized differences between the group MD and malaria alone because “real” associations would have been mixed with isolated malaria cases. It appears that co-infected patients consulted significantly later than the other groups, which may explains the predominance of IgM-diagnosed cases. However, almost all previous studies on dengue and malaria co-infections relied on IgM diagnosis [1,2,4-8,21]. Another hypothesis to explain the relatively high number of LDC in the study group is that malaria attack could have been triggered by dengue infection, especially as there is a majority of *P. vivax* infection, possibly relapses, which are coherent with the high frequency of malaria medical history, especially in the last three months in the MD [22]. As low parasitaemia was significantly more frequent in MD than in M group, another explanation could be the discovery of asymptomatic *P. vivax* infections in patients living in endemic areas, but this phenomenon has been barely described in the Amazonian region in Amerindian population, which is not the case here [23]. To ensure that low parasitaemia in the co-infection group did not result from asymptomatic infections, we tested MD patients with low parasitaemia versus D separately. The association between MD and thrombocytopaenia, anaemia, masculine gender, elevated CRP level, frequent transfusions persisted in bivariate analysis but not in multivariate analysis probably due to the loss of power. This result suggests that they were true MD co-infections with low parasite burdens.

The present study suggests an increased severity of the simultaneous infection compared to the isolated infections, which has only been hypothesized previously [1,4], in particular with haematological consequences. However, severe malaria cases as defined by the WHO were more frequent in the MD group than in M and D separately in bivariate analysis [20]. Indeed because of insufficient power, *P. vivax* and *P. falciparum* malaria were pooled, which limits the study of *P. falciparum* severe malaria. Therefore, the biological influence of dengue virus, which affects the endothelium, a major protagonist of severe malaria pathophysiology, on the eventual severity of *falciparum* malaria, needs to be studied [24].

Co-infected patients presented deep thrombocytopenia more frequently than patients with single infections. Low platelets are common in dengue and malaria. In febrile patients living or returning from endemic areas, it is a good predictive factor of malaria [25,26] and in case of negative malaria diagnosis it is a good predictive factor of dengue [25]. During malaria attack in adults, thrombocytopaenia is generally not considered to be a risk factor of haemorrhage and increased mortality [27]. Nevertheless, in non-immunized children with a malaria attack, a platelet count below 100 10^9^/L has been demonstrated to be a predictive factor of mortality [28]. Furthermore, a study performed in France on 21,888 cases of imported *P. falciparum* malaria showed that thrombocytopaenia below 50 10^9^/L was associated with an increased risk of mortality [29]. Considering dengue fever, high thrombocytopaenia is a known severity criterion and is linked to a higher mortality [30]. In the present study, severe thrombocytopaenia was not really accompanied with a recrudescence of haemorrhagic signs. No significant difference for the thrombocytopaenia between *P. vivax* and *P. falciparum* was observed. During malaria attacks, thrombocytopaenia generally worsens linearly with the increase of PL. This relationship did not clearly appear in patients co-infected with dengue so it is notable that deep thrombocytopaenia apparently occurred even with low parasite loads when associated with dengue virus.

Anaemia was more frequent in patients with dual infection. There was a convergence of indirect signs, such as pallor and transfusion need and elevated total bilirubin, probably in relation to increased haemolysis. Anaemia is a classical symptom of malaria but it is barely described in dengue fever. Indeed, elevated haematocrit is found in the severe dengue fever cases, resulting in plasma leakage syndrome [30]. The study performed on 21,888 cases of imported *P. falciparum*, showed that haemoglobin < 8 g/dL was an independent predictive factor of mortality [29].

## Conclusions

In the present study, concurrent dengue and malaria infection tends to be more severe than single infections notably for haematologic abnormalities, such as thrombocytopaenia and anaemia, known risk factors of severe dengue fever and/or malaria. However, whether this increased severity results from longer evolution duration or increased virulence or both remains to be determined. The study was retrospective so the results should be interpreted with caution. Whether prospective studies with homogeneous biological diagnosis methods and patient groups would be necessary to confirm the greatest severity of co-infection, the feasibility of such a study is questionable because of the very low prevalence of dual infection. The current evolution of these two mosquito-borne infections suggests that co-infections could become a medical problem. Since the biological and clinical characteristics of dengue and malaria are very similar, all clinicians treating patients in or returning from endemic areas should systematically order examinations for both diagnoses, even if one or the other is positive.

## Abbreviations

95 CI: 95% Confidence interval; CADC: Confirmed acute dengue cases; CRP: C-reactive protein; D: Dengue fever group; DF: Dengue fever; DHF: Dengue haemorrhagic fever; ENT: Ear, nose and throat; Ig: Immunoglobulin; IV: Intravenous; LDC: Likely dengue cases; M: Malaria group; MD: Malaria and dengue co-infection group; OR: Odds ratio; PL: Parasite load (PL); RT-PCR: Reverse transcriptase-polymerase chain reaction (RT-PCR); SGOT: Serum glutamooxaloacetate transférase; SGPT: Serum glutamopyruvate transferase; WHO: World health organization.

## Competing interests

The authors declare no competing interests.

## Authors’ contributions

LE participated in the design of the study, performed the statistical analysis, contributed to the analysis and interpretation of data and drafted the manuscript. MH performed the statistical analysis and contributed to the analysis and interpretation of data. PD carried out the the immunoassays and the RT-PCR, contributed to the analysis and interpretation of data and helped to draft the manuscript SOE contributed to conception of the study and the acquisition of data. FD conceived of the study and contributed to the analysis and interpretation of data and helped to draft the manuscript. MN conceived of the study and contributed to the analysis and interpretation of data and helped to draft the manuscript. BC conceived the study and contributed to the analysis and interpretation of data and helped to draft the manuscript. All authors read and approved the final manuscript.
